# Big data on the prevalence of injury deaths among 187 488 elderly Chinese in the past 20 years (2000–2020): a systematic review and meta-analysis

**DOI:** 10.1186/s12877-023-04056-0

**Published:** 2023-05-31

**Authors:** Shan-lan Yang, Lang-lang Zhang, Xiang Zhu, Jia-xin Tu, He-lang Huang, Chao Yu, Lei Wu

**Affiliations:** 1 Jiangxi Provincial Key Laboratory of Preventive Medicine, School of Public Health, Nanchang University, Nanchang, 330006 China; 2 Center for Prevention and Treatment of Cardiovascular Diseases, the Second Affiliated Hospital of Nanchang University, Nanchang, 330006 China

**Keywords:** Injury, Mortality, Elderly, Meta-analysis, Epidemiology

## Abstract

**Background:**

This study systematically reviewed injury death and causes in the elderly population in China from 2000 to 2020, to prevent or reduce the occurrence of injuries and death.

**Methods:**

The CNKI, VIP, Wan Fang, MEDLINE, Embase, SinoMed, and Web of Science databases were searched to collect epidemiological characteristics of injury death among elderly over 60 years old in China from January 2000 to December 2020. Random effects meta-analysis was performed to pool injury mortality rate and identify publication bias, with study quality assessed using the AHRQ risk of bias tool.

**Results:**

(1) A total of 41 studies with 187 488 subjects were included, covering 125 million elderly. The pooled injury mortality rate was 135.58/10^5^ [95%CI: (113.36 to 162.14)/10^5^], ranking second in the total death cause of the elderly. (2)Subgroup analysis showed that male injury death (146.00/10^5^) was significantly higher than that of females (127.90/10^5^), and overall injury mortality increased exponentially with age (*R*^2^ = 0.957), especially in those over 80 years old; the spatial distribution shows that the injury death rate in the central region is higher than that in the east and west and that in the countryside is higher than that in the city; the distribution of death time shows that after entering an aging society (2000–2020) is significantly higher than before (1990–2000). (3) There are more than 12 types of injury death, and the top three are falling, traffic accidents, and suicide.

**Conclusions:**

China's elderly injury death rate is at a high level in the world, with more males than females, especially after the age of 80. There are regional differences. The main types of injury death are falling, traffic, and suicide. During the 14th Five-Year Plan period, for accidental injuries and death, a rectification list for aging and barrier-free environments was issued.

**PROSPERO Registration:**

The systematic review was registered in PROSPERO under protocol number CRD42022359992.

## Introduction

The proportion of the population over 60 years old is up to 18.7% according to the 7th National Census of China (2020) [1], which means that China has entered a deep aging society. In 2021, the National Bureau of Statistics of China reported that the total number of deaths in the country reached nearly 10.14 million, of which more than 2/3 of the population is over 60 years old [2]. The top three causes of death in China are chronic diseases, injuries, as well as infectious diseases, maternal and infant diseases, and nutritional deficiencies, with injury-related deaths ranking second among the elderly [3]. In addition to death, disability, and personal and family suffering, the consequences of injury will also cause a serious burden of medical care [4]. In this regard, the Chinese Communist Party and government are highly concerned about the death of elderly injuries and have introduced relevant strategies and measures. However, research on injury death of the elderly is still mostly limited to a certain province, prefecture, and city, and there is no comprehensive analysis of the situation In this systematic review, we use big data to conduct a meta-analysis on the types of injury deaths, the characteristics of "population, places, and times" and dynamic changes in the elderly population in China in the past 20 years (2000–2020), and the results can provide a basis for formulating corresponding prevention and control measures, thereby preventing and reducing the occurrence of injuries and deaths.

## Material and methods

The systematic review was registered in PROSPERO under protocol number CRD42022359992.

### Inclusion and exclusion criteria

Inclusion criteria: (1) Resident senior citizens aged 60 years in China from 2000 to 2020; (2) Injury death as the research outcome goal; (3) Cause of death classified according to ICD-10 code; (4) Provide injury mortality or the number of deaths, sample size, or the required value can be calculated based on the data given in the literature; (5) Source of data from the local death cause monitoring system or authoritative system; (6) Cross-sectional study.

### Outcomes

#### Injury mortality among the elderly

Exclusion criteria: (1) Documents with ambiguous, incomplete, or unconverted data; (2) The quality of the literature is too low (AHRQ score below 3); (3) Repeated publications.

### Search strategy

This review was conducted according to the Preferred Reporting Items for Systematic Reviews and Meta-Analyses (PRISMA) guidelines and was searched through seven electronic databases without language restriction: Chinese National Knowledge Infrastructure (CNKI), Chongqing VIP Database, WanFang Database, MEDLINE, Embase, SinoMed and Web of Science. To collect epidemiological cross-sectional research papers on injury and death of Chinese people aged ≥ 60 years published in China and abroad, and the search time limit was from January 2000 to December 2020 (including references to the literature to supplement relevant studies). The retrieval takes the combination of subject headings and free words. Chinese search terms include: “中国”, “中国人”, “我国”, “老年人”, “老人”, “老年”, “伤害死亡”, “伤害”, “死因”, “死亡率”, “粗死亡率”, “标化死亡率”, “死因监测”; English search terms include China, Chinese, elderly, elderly people, aged, injury death, injury, death, mortality rate, crude mortality rate, standardized mortality rate, mortality surveillance.

### Literature screening and data extraction

All publications that were identified from literature searches were initially extracted by two independent reviewers. Data were stored in a standardized tabular format and the full list was assessed for eligibility by two different reviewers independently. Following the screening, any discrepancies were discussed between the reviewers. Any further conflicts were resolved by reviewing the original publication and additional adjudication. Information from selected literature was extracted as followed: (1) The first author, the area, and time of the investigation; (2) Outcome indicators (injury mortality) and outcome measurement data (sample size, age and gender of patients, place of residence, investigation time and injury type); (3) Key elements of risk assessment for bias.

### Quality assessment

Evaluation tools recommended by *Agency for Healthcare Research and Quality* (AHRQ) tool(www.ahrq.gov) were used to assess the quality of all cross-sectional studies. AHRQ was an 11-item instrument with 3 options: the ‘Yes’ would be scored as ‘1’, ‘No’ or ‘Unclear’ would be scored as ‘0’. The articles were scored as follows: 0–3 = low quality; 4–7 = moderate quality; 8–11 = high quality. The quality of the literature was evaluated by two independent reviewers (Y.SL and T.JX), and a third party was consulted in case of disagreement.

### Statistical analysis

All results for the prevalence of injury mortality among 187 488 elderly Chinese were visualized using forest plots. The heterogeneity between the involved studies was assessed using the *I*^*2*^ statistic and *Q* tests. The fixed effects model was used when the heterogeneity test results were considered to be good(*P* > 0.1 and *I*^*2*^ < 50%); conversely, the random effects model was utilized when *I*^*2*^ ≥ 50%. Subgroup analysis was carried out according to the characteristics of the population, such as gender, age, region, urban and rural distribution, statistical year, and injury type; sensitivity analysis was carried out by one-by-one elimination method, and Publication bias was assessed using Egger's test and a funnel plot of linear regression of the log ORs on the inverse root of the sample size, and *P* < 0.05 was considered statistically significant difference. Statistical analysis was performed with the software R (R Project for Statistical Computing, version 4.1.2, http://www.r-project.org/).

## Results

### Literature screening process and results

**Fig. 1:**
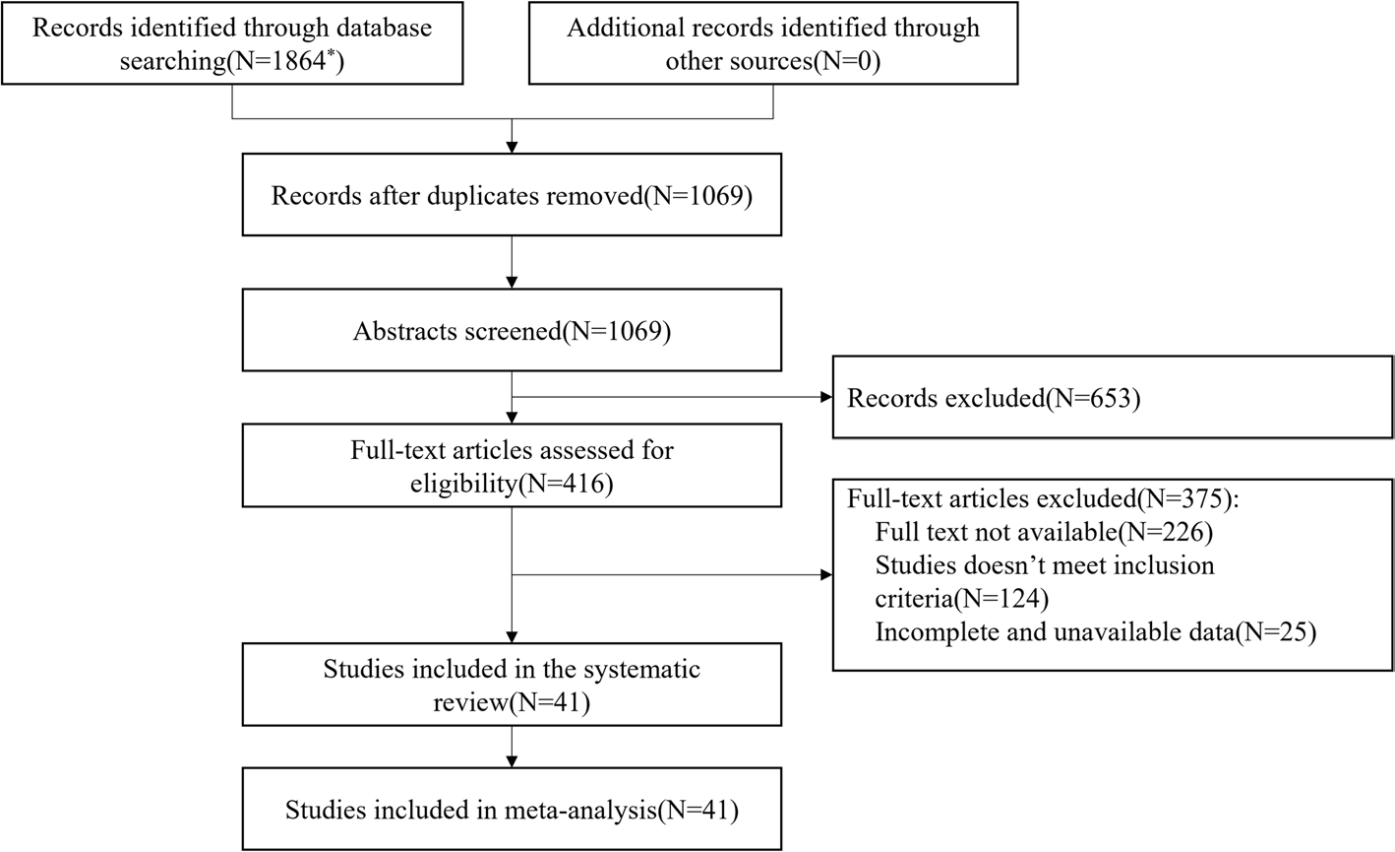
Flow diagram for systematic review and meta-analysis. Flowchart demonstrating selection of eligible studies. The databases retrieved and the number of the literature identified are as follows: CNKI (*N* = 91), VIP (*N* = 63), WanFang Data (*N* = 389), MEDLINE (*N* = 193), SinoMed (*N* = 13), Embase (*N* = 3), and Web of Science (*N* = 1112)

A total of 1864 related pieces of literature were originally identified, and after the layer-by-layer screening, 41 pieces of literature were ultimately incorporated, with a total of 187 488 elderly people who died of injury, comprising a sample population of 124 798 756. The literature screening process and results are shown in Fig. 1.

### Basic characteristics and risk of bias assessment of the included literature

The basic characteristics and risk of bias evaluation of the incorporated literature are demonstrated in Table 1.

**Table 1:** Main information and risk of bias assessment of the included literature

Author	Journal IF and Type		Year of publication	Location	Total N	Injury fatalities N	Gender (Male/Female N)	Injury mortality / 10^5^	AHRQ score
Teng YM [5]	1.02		2021	Guangxi /	5 488 375	8451	4809/3642	153.98	7
Wei XL [6]	1.58	√_1_√_2_√_3_	2020	Jiangsu U	14 625 370	23 177	1 0621/1 2556	158.47	7
Li ZK [7]	2.84	√_1_√_2_√_3_	2020	Yunnan U	9 502 667	12 114	6843/5271	127.48	7
Liao ZY [8]	1.51	√_3_	2020	Sichuan U	915 600	1317	731/586	143.84	7
Zhang CH [9]	1.98	√_3_	2020	Chongqing /	18 617 361	26 230	14 901/11 329	140.89	7
Huang LL [10]	1.56	√_3_	2020	Guangdong U	852 219	816	392/424	95.75	7
Xu C [11]	1.81	√_3_	2020	Sichuan R	353 759	448	/	126.64	7
Shi AY [12]	1.27		2019	Shanghai U	1 760 189	2613	1241/1372	148.45	7
Yu X [13]	2.15	√_1_√_2_√_3_	2019	Beijing U	1 675 901	1084	/	64.68	7
Zeng C [14]	2.84	√_1_√_2_√_3_	2019	Guangdong U	583 022	375	/	64.32	7
Huang CY [15]	2.84	√_1_√_2_√_3_	2018	Jiangsu U	18 804 009	28 332	13 155/15 177	150.67	8
Xiang YF [16]	0.76		2018	Zhejiang /	696 443	1723	/	274.40	8
Gong HY [17]	1.98	√_3_	2017	Hubei U + R	9 829 478	21 159	10 566/10 593	215.26	7
Zhang XY [18]	1.86	√_1_√_2_√_3_	2017	Shanxi U	4 149 374	5039	3036/2003	121.44	7
Zhang DK [19]	1.01		2017	Shandong U	185 865	329	173/156	177.01	7
Wang HF [20]	1.02		2016	Shandong U	925 672	1081	654/427	116.78	6
Dong H [21]	2.37	√_3_	2016	Guangdong /	2 594 725	2052	1030/1022	79.08	7
Mu SC [22]	2.03	√_1_√_3_	2016	Shandong U	252 873	356	209/147	140.78	7
Liu ZH [23]	2.03	√_1_√_3_	2015	Shandong R	338 156	402	/	118.88	7
Cai P [24]	2.15	√_1_√_2_√_3_	2014	Zhejiang U + R	1 756 853	5723	2945/2778	325.75	7
Wan QP [25]	2.74	√_1_√_3_	2014	Shanghai /	789 094	1039	408/631	131.67	7
Qin L [26]	1.86	√_1_√_2_√_3_	2014	Liaoning /	599 138	459	/	76.61	7
Bi SF [27]	1.43		2014	Neimenggu /	1 618 047	1370	/	84.67	7
Chen HJ [28]	1.27		2013	Zhejiang R	486 555	1298	643/655	266.77	6
Li L [29]	2.15		2013	Zhejiang /	6 171 228	10,214	/	165.51	7
Ying JW [30]	2.02	√_1_√_2_√_3_	2013	Zhejiang R	266 257	687	/	258.02	6
Shao YQ [31]	2.15	√_3_	2012	Zhejiang /	678 180	1727	863/864	254.652	7
Wang QQ [32]	2.02	√_1_√_2_√_3_	2012	Zhejiang R	242 870	849	/	349.57	7
Mei QH [33]	2.15	√_3_	2011	Zhejiang U + R	7 586 056	14 542	6895/7647	191.69	7
Xu HF [34]	1.34	√_1_√_2_√_3_	2011	Guangdong U + R	1 756 981	2576	1333/1243	146.6	7
Zhang YH [35]	1.02	√_3_	2011	Jiangsu /	2 026 492	2261	/	111.57	7
Xie HY [36]	1.34	√_3_	2011	Guangdong U + R	1 748 946	2576	1330/1246	147.29	6
Liang H [37]	1.70	√_3_	2010	Guangxi R	97 493	175	109/66	179.5	6
Zhou L [38]	1.44		2008	Shanghai U	903 100	1069	422/647	118.37	7
Gong HQ [39]	1.18	√_1_√_2_√_3_	2008	Yunnan U + R	480 301	952	579/373	198.21	5
Bai HZ [40]	1.27	√_3_	2008	Shanghai U	4 174 330	602	259/343	15.34	7
Ni JH [41]	1.58		2008	Shanghai /	3 047 527	986	391/595	32.35	7
Peng YY [42]	2.03	√_3_	2007	Shanghai U	552 208	559	326/233	101.23	7
Hu XQ [43]	1.54	√_1_√_3_	2007	Zhejiang /	110 313	298	135/163	270.14	7
Yang JZ [44]	1.89	√_3_	2006	Guizhou U	91 105	197	/	216.23	7
Sun XK [45]		√_1_√_2_√_3_	2006	Jiangsu /	164 624	231	120/111	140.32	7

Note: ①√_1_ is Chinese core journals, √_2_ is Chinese Science Citation Database, √_3_ is China Science and Technology Papers and Citation Database;

②/ = Not described; *U *Urban, *R *Rural

## Results of the meta-analysis

### Literature basic situation and quality

**Fig. 2:**
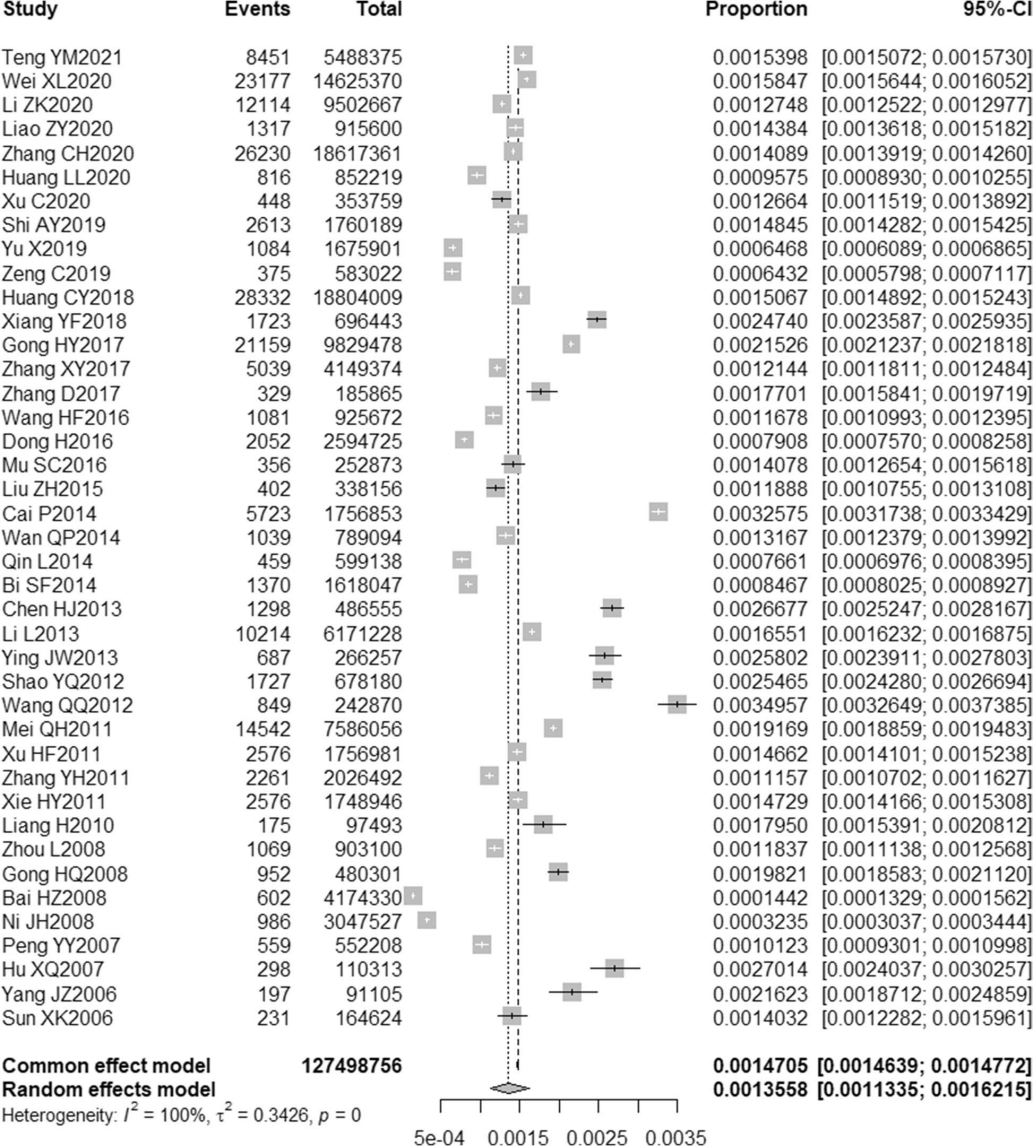
Forest plot of injury mortality point values and interval estimates

This review collected 41 pieces of literature, and the average AHRQ score was 6.85, demonstrating that the quality of the literature was optimal. The sample involved 124 798 756 elderly people, with 187 488 injury death, in which males (85 119) were slightly more than females (82 300). According to the results of the heterogeneity test (*P* < 0.001, *I*^2^ = 100%), a single-sample rate meta-analysis was performed using a random-effects model, and the results of the forest plot are demonstrated in Fig. 2.

### Total injury mortality

The results of the random effects model showed that the total injury mortality of the elderly in China was 135.58/10^5^ [95%CI: (113.36 to 162.14)/10^5^].

### ***Subgroup analysis Results (1/10***.^***5***^***)***

**Fig. 3:**
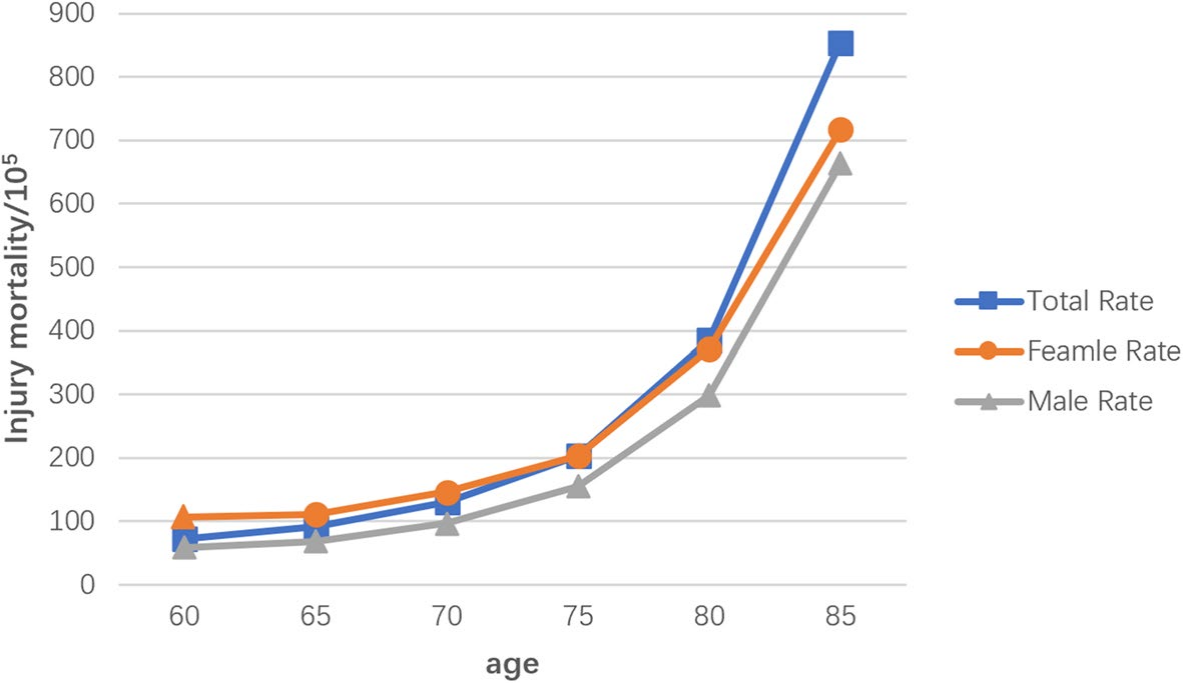
Changes in injury mortality rates among the elderly of different age groups in China, 2000–2020

**Fig. 4:**
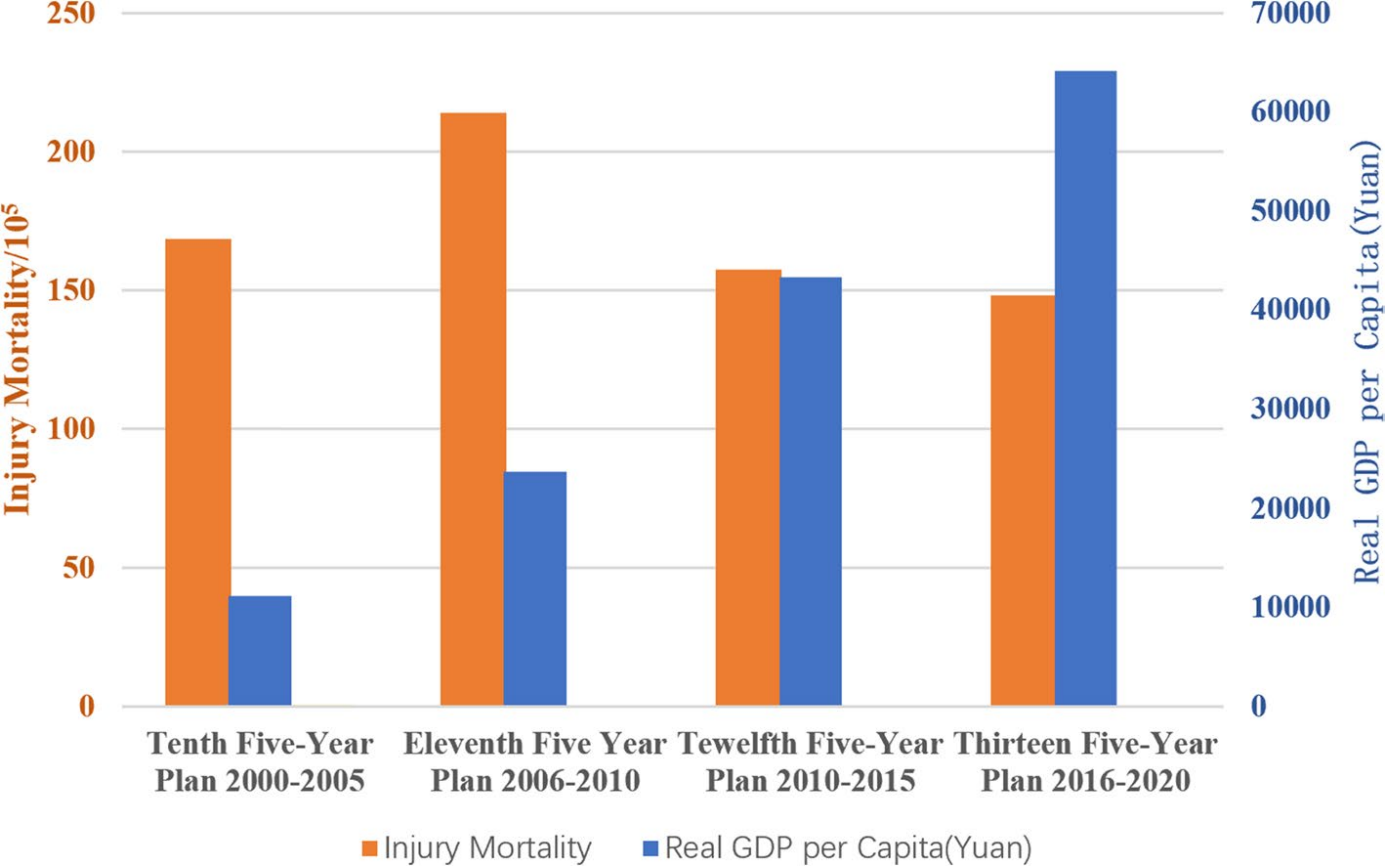
Columnar diagram of GDP level and elderly injury mortality during the four five-year plans

**Fig. 5:**
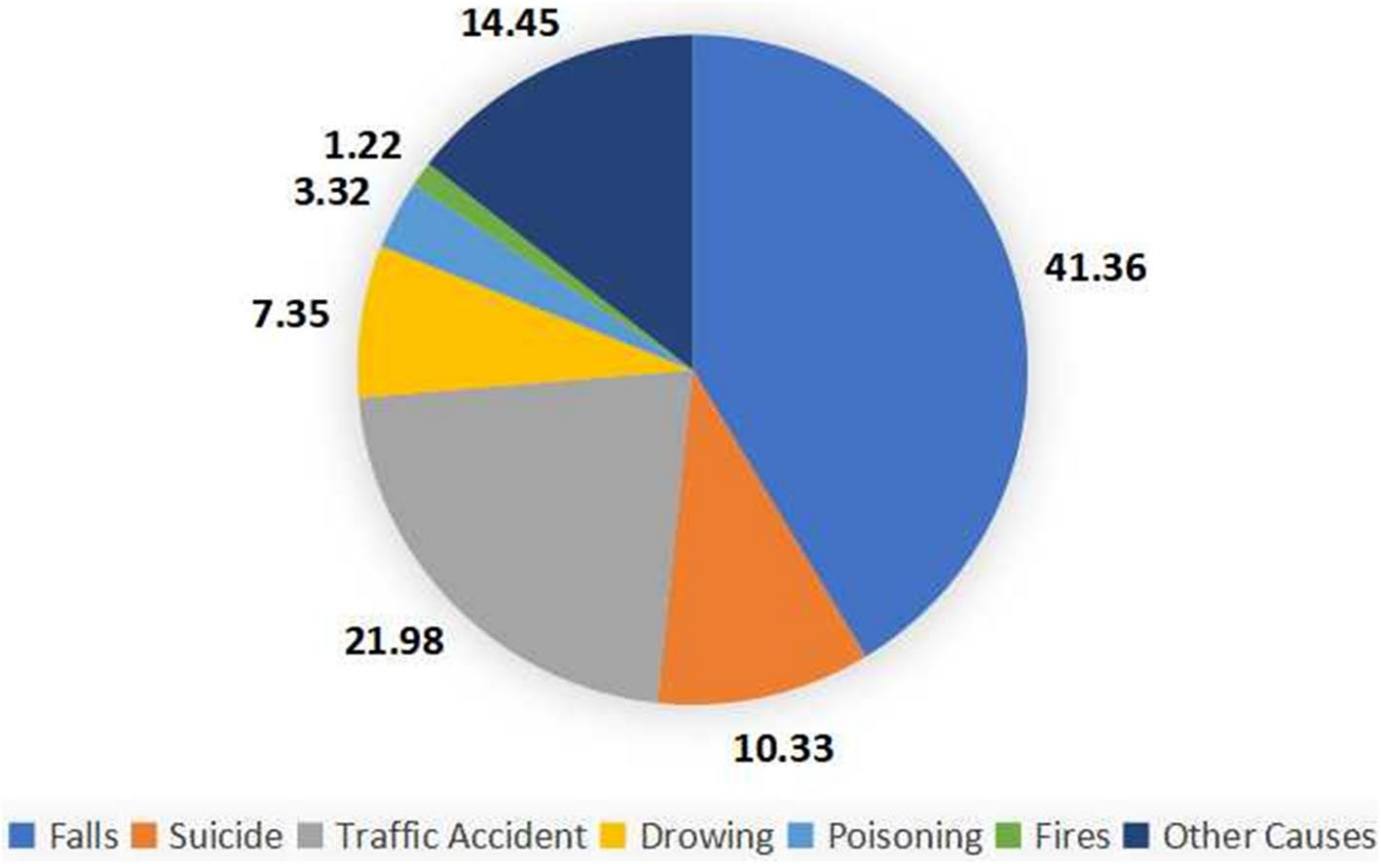
Composition of different injury causes of death among Chinese elderly from 2000 to 2020 (%)

The subgroups analyzed the injury mortality rate of different factors such as gender, age, region and time. Due to the large heterogeneity among subgroups, random model analysis was still used. The results showed that the mortality rate of males was higher than that of females (146.00 vs. 127.90, *P* < 0.05); the author divided the age into six groups, and the mortality rate increased sharply with age, especially in the group over 80 years old (60-year-old: 72.01 < 65-year-old: 91.83 < 70-year-old: 130.05 < 75-year-old: 203.25 < 80-year-old: 384.88 < 85-year-old: 854.42, *P* for trend < 0.05), see Fig. 3 and Table 2; according to the spatial analysis, the mortality rate in China was as follows: middle: 215.26 > western: 143.84 > east: 130.73, rural (170.35) is higher than urban (131.99); in terms of time, the injury mortality rate was calculated based on the per capita GDP level for 20 years (across four five-year plans), In the first two five-year plans, the overall change demonstrated the same trend as the per capita GDP level, although in the latter two, the trend was the opposite (Fig. 4). According to 41 included literatures, more than 12 types of injuries were involved, and the top three were falling (43.91), traffic accidents (24.49), and suicide (15.88), see in Table 3; and the composition of different causes of death from injuries among the elderly in China from 2000 to 2020 (%) is shown in Fig. 5.

**Table 2:** Random effects model subgroup analysis and comparison

Characteristic	Population	Injury fatalities	Literature number	Heterogeneity		Injury mortality(95%CI) /10^5^
				***P***	***I***^**2**^**%**	
**Total**	124,798,756	187,488	41 [5–45]	0.0000	100	135.58(113.36,162.14)
**Sex**						
Male	53,548,047	84,793	28 [5–10, 12, 15, 17–22, 24, 25, 28, 31, 33, 34, 36–41, 43, 45]	0.0000	99.7	146.00 (116.00,183.74)
Female	58,613,602	82,067	28 [5–10, 12, 15, 17–22, 24, 25, 28, 31, 33, 34, 36–41, 43, 45]	0.0000	99.7	127.90 (102.31,159.88)
**Age**						
60 ~	12,678,447	9130	8 [6, 8, 17, 21, 22, 24, 33, 35]	0.0000	99.3	72.01 (70.55,73.50)
65 ~	11,250,673	10,332	10 [5, 6, 8, 17, 19, 21, 22, 24, 33, 35]	0.0000	98.8	91.83 (90.08,93.62)
70 ~	8,509,655	11,067	10 [5, 6, 8, 17, 19, 21, 22, 24, 33, 35]	0.0000	99.4	130.05 (127.65,132.50)
75 ~	6,481,340	13,173	10[﻿^5^, ^6^, ^8^, ^17^, ^19^, ^21^, ^22^, ^24^, ^33^, ^35^]	0.0000	99.6	203.25(199.81,206.74)
80 ~	3,943,769	15,179	10 [5, 6, 8, 17, 19, 21, 22, 24, 33, 35]	0.0000	99.7	384.88 (378.82,391.04)
85 ~	2,397,524	20,485	10 [5, 6, 8, 17, 19, 21, 22, 24, 33, 35]	0.0000	99.7	854.42 (842.85,866.15)
**Area**						
Middle China	9,829,478	21 159	1 [17]	/	/	215.26 (212.38,218.18)
Eastern China	76,355,196	110,036	30 [6, 10, 12–16, 19–26, 28–36, 38, 39, 41–43, 45]	< 0.0000	100	130.73 (103.20,165.60)
Western China	41,314,082	56,293	10 [5, 7–9, 11, 18, 27, 37, 40, 44]	< 0.0000	100	143.84 (122.80,168.46)
**Urban/rural**						
Urban	88,852,752	130,789	23 [6–10, 12–15, 17–20, 22, 24, 33, 34, 36, 38–40, 42, 44]	0.0000	99.9	131.99 (101.85,171.05)
Rural	14,931,279	26,225	12 [11, 17, 23, 24, 28, 30, 32–34, 36, 37, 39]	0.0000	99.8	170.35 (134.05,216.45)
**Years**						
2000 ~ 2005	8,293,637	13,889	8 [33, 34, 36, 39, 42–45]	0.0001	97.2	168.45(149.95,189.23)
2006 ~ 2010	14,004,751	27,143	9 [6, 7, 17, 18, 24, 31–33, 37]	0.0001	99.2	213.96 (184.75,247.76)
2011 ~ 2016	38,631,767	61,930	11 [5–9, 16–19, 21, 22, 24]	0.0001	99.6	157.18(139.52,177.07)
2016 ~ 2020	19,963,976	30,397	6 [5–9, 11]	0.0001	97.6	148.10 (138.13,158.79)

**Table 3:** Combined rates of injury deaths by region, gender, and type of injury in China in this study

Type	Total		Area				Gender			
	**Mortality rate (95%CI)/10**^**5**^	**Composition ratio (%)**	**Eastern China**		**Western China**		**Male**		**Female**	
			**Mortality rate (95%CI)/10**^**5**^	**Composition ratio (%)**	**Mortality rate (95%CI)/10**^**5**^	**Composition ratio (%)**	**Mortality rate (95%CI)/10**^**5**^	**Composition ratio (%)**	**Mortality rate (95%CI)/10**^**5**^	**Composition ratio (%)**
Falling	43.91 (34.38,56.06)	41.36	48.05 (37.27,61.95)	39.59	33.72 (16.76,67.85)	38.80	41.93 (30.70,57.26)	32.94	47.63 (36.00,63.00)	45.24
Traffic accident	24.49 (19.52,30.72)	21.98	23.91 (18.59,30.75)	22.97	22.93 (13.51,38.91)	22.26	30.62 (22.11,42.41)	30.64	15.84 (11.31,22.19)	17.12
Suicide	15.88 (11.30,22.33)	10.33	12.98 (9.10,18.52)	8.70	16.83 (7.17,39.50)	13.50	13.72 (8.71,21.61)	9.74	11.81 (7.47,18.66)	8.12
Drowning	6.26 (4.42,8.87)	7.35	5.96 (3.90,9.10)	9.06	6.53 (3.75,11.35)	6.42	5.91 (4.04,8.64)	7.76	5.96 (3.88,9.16)	10.58
Poisoning	4.43 (3.18,6.18)	3.32	3.86 (2.65,5.63)	2.04	7.68 (4.71,12.52)	3.46	4.95 (2.72,8.99)	2.64	3.31 (1.89,5.79)	1.46
Fires	2.26 (1.50,3.39)	1.22	2.09 (1.38,3.16)	1.21	3.32 (2.79,3.96)	0.72	2.86 (1.94,4.22)	1.28	1.63 (1.18,2.26)	0.90
Other causes	14.33 (9.61,21.36)	14.45	14.56 (7.41,28.61)	16.42	10.41 (5.71,19.00)	14.85	15.45(9.84,24.27)	14.99	13.47 (8.19,22.16)	16.04
Total	127.98 (105.20,155.69)	100.00	129.23 (105.17,158.79)	100.00	107.88 (62.11,187.31)	100.00	126.18 (93.55,170.18)	100.00	112.33 (84.65,149.06)	100.00

Due to the limitation of data integrity, the number of included papers is different, and there are differences between Tables 3 and 2 in the eastern, western, and male and female combined rates, and Table 2 shall prevail

According to the statistics from 41 literatures, there are more than 12 causes of injury and death in China. They are falling, traffic accidents, suicide, drowning, poisoning, fire, homicide, transportation accidents, electric shock, crushing, accidents caused by the natural environment, suffocation, etc. The first six types accounted for 85.55% of the total causes of death. Falling is the main cause of death (41.36%), followed by traffic and suicide. The death rate of the first two injury types was higher in eastern than in western, and the fall death rate for males was slightly lower than that of females, but the suicide death rate was slightly higher than that of females, and the traffic death rate was 1.79 times higher than that of females.

### Sensitivity analysis

**Fig. 6:**
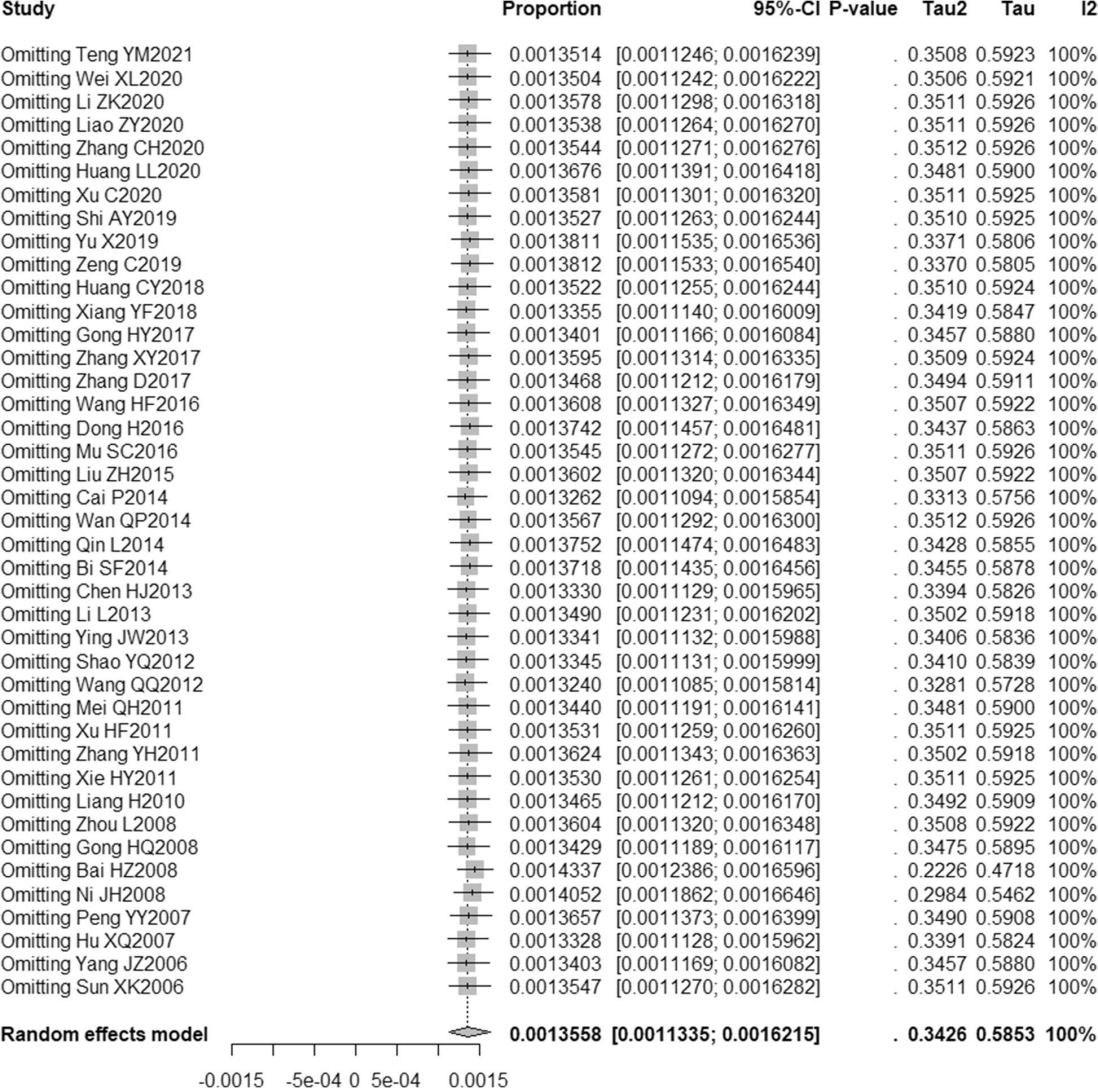
Forest map after culling one by one

The sensitivity analysis was carried out by the one-by-one elimination method, and the results were between 132.40/10^5^ and 143.37/10^5^, which was not much different from the total injury mortality rate, indicating that the results of this study were stable, as shown in Fig. 6.

### Evaluation of publication bias

**Fig. 7:**
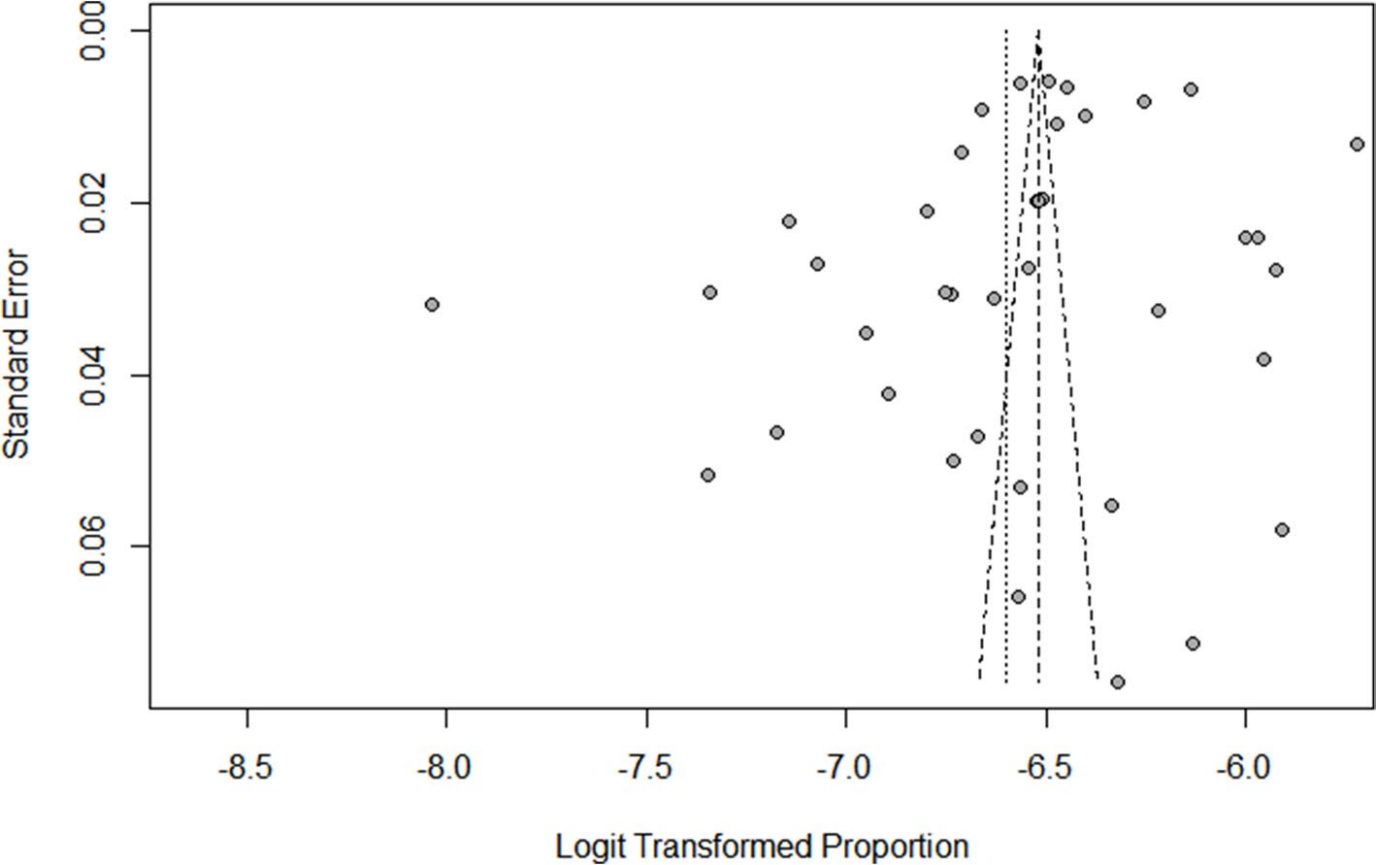
Funnel plot of total injury mortality rate

The funnel plot of total injury mortality was not significantly asymmetric. Combined with the results of Egger's plot (*t* = -1.58, *P* = 0.122), it suggested that there was a small possibility of publication bias, see Fig. 7.

## Discussion

The above results show that the injury mortality rate of the elderly in China after entering the aging period is higher than that before and has broken the original stable situation [46]; it is higher than that of the United States and Brazil in 2000 and is at a medium–high level compared with the EU countries in the early twenty-first century [47–49]. Mortality levels indicate that injury mortality in the elderly population varies by country and time. Of course, to more accurately compare this rate with countries, a standardized rate is ideal.

Another prominent feature of injury death in China is that there is a gender difference between males and females, which is consistent with the results of most countries. The mortality rate of different age groups varies significantly. Injuries increase exponentially with age and the rate of falling increases with age. The reason may be the negative impact of aging on the elderly [50], and it is particularly important to prevent falling. The world has become intelligent, and the elderly are not as receptive to intelligent technology as the young and middle-aged. For example, in bad weather, a young man can use his smartphone to solve his lunch while sitting in an office, while an old man needs to go to the street to buy food. It is entirely possible to suffer injury.

2000–2020 was the period from the implementation to the completion of China's four "five-year plans", during which China completed the rapid change from reform to opening up, and the Chinese economy, social environment, family living patterns, and even development policies changed dramatically. This has resulted in large differences in economic development and GDP levels between central, eastern, and western China, which inevitably affect the overall changes in injury mortality among older adults in China. In the first decade, the flow of economic growth led to frequent population movements and traffic, and these phenomena inevitably resulted in a relative increase in traffic accidents and other accidental injuries [51]. GDP growth is largely dependent on industrial production and rising labour levels, which have resulted in increased stress in work and life; these physical to psychological stresses have been reported in other studies in China, so their effects on accidental injuries cannot be ruled out either. GDP growth has a positive impact on increasing the country's economic power, improving people's living standards, and promoting social progress. Meanwhile, from a management and public health perspective, it may play a significant role in reducing the occurrence and prognosis of injuries if the positive effects of GDP are adequately considered.

In the early stage of China’s reform and opening up, the “Seventh Five-Year Plan” divided the entire region into eastern, central, and western regions according to the high, intermediate, and low levels of economic and technological development [52]. The injury mortality in the central region is the highest, which can be explained from the following three aspects. First, compared with the eastern and coastal regions, central China has a cold climate, relatively scarce resources, and relatively lagging economic level [53, 54]. Moreover, diet type and structure, lifestyle, and habits differ among the regions (such as Inner Mongolia, Heilongjiang, Jilin Province, etc.), and the increased risk of death from accidental injuries cannot be excluded as a long-term effect of these factors. Second, the social security development system and speed in the central region are relatively lagging, and the medical and healthcare technology and facilities for elderly special groups are not as ideal as those of the eastern or coastal regions [55]. Lastly, the density of transportation arteries in central China (such as Jiangxi, Hubei, Hunan Province, etc.) is relatively high [56], and many transportation lines are designed to pass through dense areas of urban dwellers due to the regional culture and lifestyle mindset, which to a certain extent are more prone to traffic accidents. While the injury mortality in the eastern region is slightly higher than that in the western region. It is believed that this phenomenon does not rule out the double-edged sword effect of factors such as the aging degree, the higher GDP level, and the more congruent health care system in the eastern region.

After the urbanization development was included in the special key plan in China's "Tenth Five-Year Plan", the level of urbanization has climbed sharply over the past 20 years, from 36% in 2000 to 63.89% in 2020 [2]. This trend has brought about major changes in the rural population, labor force size, and structure –– the average age of agricultural laborers is 48.45 years old, and as many as 75.24% are female. The influx of rural youth and middle-aged labor into cities has considerably accelerated the aging and feminization of the rural population [57], which is also one of the factors that cause rural injuries to be higher than urban ones.

Another feature is that injury mortality in China has raised firstly and then reduced over the past 20 years (2000–2020). Since entering an aging society in 2000, the injury mortality rate has remained high for ten years. With the emergence and popularization of the concept of population aging, the national injury prevention, and control work has been carried out in an all-round way [58], and the Chinese Party and government have issued the "Quality Development Outline (2011–2020)", "National Disability Prevention Action Plan (2016–2020)", "Healthy China 2030 Planning Outline" and a series of strategies or measures such as injury prevention and control, such as road safety-related policies, although the activities of injury prevention, obstacle reduction, and aging-appropriate renovation have been carried out at different levels in China, and achieved certain results, the degree of aging in China Unabated, resulting in a continued increase in injury mortality.

The composition of the cause of injury death still displayed certain characteristics. In terms of period, the top six causes of death in China, including falling, traffic accidents, and suicide, cover 84.1% of the total injury causes, which is substantially higher than that in the EU (64.1%). Among these causes of death, falling is the earliest cause of injury death. Among Chinese people aged 65, an average of 3–4 people in every 10 have fallen. According to this estimate, about 57.192 million to 76.256 million people will fall every year at this stage, and this phenomenon will increase with the acceleration of the aging degree. 40% to 70% of people who fall will suffer from various degrees of injury and receive medical treatment. It is worth mentioning that about 22.34% of those who fall have suffered serious injury or even paralysis [59].

In contemporary China, traffic accidents have virtually become a social problem. Since the beginning of the twenty-first century, the mileage of highways and the number of motor vehicles have raised markedly and have been extensively managed. Road traffic injuries are extremely common, among which injuries involving "people" account for 78.3% ~ 96.5% of all causes, and illegal acts such as speeding are very common [60]. At the same time, frequent and dense population flow leads to traffic jams and traffic accidents.

Compared with the BRICS countries (China, Russia, Brazil, India, and South Africa), the suicide mortality rate among those aged 70 years is the highest in China, and the phenomenon of suicide is severe (2015) [61]. The main reasons for this phenomenon are: first, illness and persistent economic burden. The prevalence of chronic diseases among the elderly in China is 86.23%, of which 76.30% suffer from various chronic diseases at this stage [62, 63]; the second is lack of emotion and loneliness. If it cannot be ruled out that China's "family planning" policy, which was set as a basic national policy in 1982, has led to the emergence of a large number of "4 + 2 + 1 Family", "8 + 2 + 1 Family" and " The Loss of Only Child Family", factors such as widowhood can affect emotions [64].

Because of the current situation of injury and death among the elderly in China, during the "14th Five-Year Plan" period, a list of rectifications such as suitable aging and a barrier-free environment has been issued. However, to achieve Healthy Aging, multiple aspects need to be integrated, such as industrial structure adjustment, improving the elderly's Self-health quality, creating a humanistic environment of government-community-family integration, and reducing the risk of injury and death.

This study has certain limitations. First, the heterogeneity of the included studies is high due to the characteristics of a single rate meta-analysis; second, in the hierarchical analysis, the literature data from different regions are not uniform, and it is also difficult to obtain data standardization background information; third, the analysis of the influencing factors of accidental death is limited to a certain extent, and there is no data on living habits, psychological factors, and post-injury nursing behaviors.

This systematic review objectively reflects the current status of injury deaths and causes of death among the elderly in China. The data is derived from the public security bureaus, death-cause monitoring sites, and CDCs of various provinces and cities in China, which are authentic and authoritative. Sensitivity analysis showed that the results of the meta-analysis of this study were stable, and the publication bias assessment also showed that there was no obvious publication bias, so the results obtained were more representative and reliable than a single study. The results of this study provide an important foundation for future prospective research and the initiation of advanced injury prevention plans in China. Governments and local institutions should consider implementing public health measures to prevent injury death. Examples include reducing income inequality, allocating more resources to vulnerable groups, and implementing targeted group prevention [65].

## Data Availability

The datasets generated and/or analysed during the current study are not publicly available due this is a systematic review and meta-analysis of publishing and fully randomized studies but are available from the corresponding author on reasonable request.

## Abbreviations

AHRQ: Agency for Healthcare Research and Quality
EU: European Union
CDC: Center for Disease Control and Prevention
